# The impact of the housing crisis on self-reported health in Europe: multilevel longitudinal modelling of 27 EU countries

**DOI:** 10.1093/eurpub/ckw071

**Published:** 2016-05-23

**Authors:** Amy Clair, Aaron Reeves, Rachel Loopstra, Martin McKee, Danny Dorling, David Stuckler

**Affiliations:** 1 Department of Sociology, University of Oxford, United Kingdom; 2 Department of Public Health and Policy, London School of Hygiene & Tropical Medicine, London, United Kingdom; 3 School of Geography and the Environment, University of Oxford, United Kingdom

## Abstract

**Background**: Many EU nations experienced a significant housing crisis during the Great Recession of 2008–10. We evaluated the consequences of housing payment problems for people’s self-reported overall health. **Methods**: We used longitudinal data from the EU Statistics on Income and Living Conditions survey covering 27 countries from 2008 to 2010 to follow a baseline sample of persons who did not have housing debt and who were employed (45 457 persons, 136 371 person–years). Multivariate linear regression and multilevel models were used to evaluate the impact of transitions into housing arrears on self-reported health, correcting for the presence of chronic illness, health limitations, and other potential socio-demographic confounders. **Results**: Persons who transitioned into housing arrears experienced a significant deterioration in self-reported overall health by − 0.03 U (95% CI − 0.01 to − 0.04), even after correcting for chronic illness, disposable income and employment status, and individual fixed effects. This association was independent and similar in magnitude to that for job loss (−0.02, 95% CI: −0.01 to − 0.04). We also found that the impact of housing arrears was significantly worse among renters, corresponding to a mean 0.11 unit additional drop in health as compared with owner-occupiers. These adverse associations were only evident in persons below the 75th percentile of disposable income. **Discussion**: Our analysis demonstrates that persons who suffer housing arrears experience increased risk of worsening self-reported health, especially among those who rent. Future research is needed to understand the role of alternative housing support systems and available strategies for preventing the health consequences of housing insecurity.

## Introduction

Since 2008, there has been widespread concern that economic uncertainty generated by the Great Recession would pose risks to public health.^1^ Much research has focused on the consequences of unemployment and job insecurity for mental health.^2^^,^^3^ Relatively less attention has been paid to the impact of people’s housing difficulties, even though the crisis had its origins in the troubled housing sector.^4^ Across Europe 4.0% of people experienced housing debt in 2010, up from 3.3% in 2008 (EU 27), corresponding to around 3.5 million people falling into arrears in this period. Despite signs of economic recovery in Europe, the proportion of persons facing housing difficulty remains above prerecession levels.

Housing has long been recognized as a determinant of health.^5^ Historically, epidemiological studies focused on how the physical attributes of housing such as damp, mould and cold could increase risks to asthma and child survival, among other outcomes.^6^ More recently, particularly with the onset of the Great Recessions in Europe, there has been interest in the consequences of eviction and homelessness, particularly on mental health in the USS and UK.^3^^,^^7–12^ A cross-state US analysis found that home foreclosures were associated with increased hospital visits for preventable conditions as well as heart attacks, strokes, and urinary tract infections.^10^ One qualitative study, using 14 focus groups among low- and moderate-income homeowners threatened with foreclosure found elevated risks of stress, anxiety and relationship strain, as well as medical debt, with potential future consequences for accessing health care.^11^ There is also evidence that high housing costs can crowd-out expenditure on health and are a major cause of personal debt, itself associated with mental disorders.^13^^,^^14^

Despite the magnitude of the housing crisis in Europe there is a relative dearth of research evaluating the health impact of housing insecurity, particularly cross-nationally and longitudinally. Most have focused on single country cases and rely on cross-sectional data.^10^^,^^11^^,^^15^^,^^16^ Importantly these studies fail to address what might be termed a ‘healthy housing effect’, analogous to the healthy worker effect, such that persons who experience financial problems associated with housing may have experienced other financial difficulties that would pose a risk to health irrespective of insecure housing. Additionally, with one important exception,^17^ they have failed to differentiate impacts on owners and renters, whose circumstances differ considerably and likely modify the effects of housing insecurity. When compared with owners, renters tend to have smaller savings, including a lack of equity associated with home ownership, which may render housing hardship more dangerous.^17^^,^^18^

In this study, we draw on longitudinal data from the EU Statistics on Income and Living Conditions (EU-SILC) to compare the health consequences for people across Europe who transition into housing arrears. We adopted a cross-national approach for two main reasons. First, the housing crisis was a pan-European phenomenon. This creates a source of quasi-exogenous variation as the economic crisis impacted the entire housing sector across countries thereby increasing the risk of arrears independent of individual factors that may confound the relationship between housing payment problems and health and creating variation in the exposure that reduces potential confounding by individual level factors. Second, the cross-European analysis makes it possible to describe the consequences of housing insecurity across the region, consistent with the national basis of public health policy. Specifically, we test the hypothesis that the consequences of housing arrears would be worse in persons who live in rented accommodation than for those living in owner occupied housing.

## Methods

### Data source

We use the 2010 longitudinal EU-SILC dataset, a nationally representative comparative survey of EU member states. The EU-SILC has been described elsewhere; briefly, it uses a 4-year rotational sample in which 25% of the sample is replaced in each year (the precise means of collection varies across countries).^19^^,^^20^ Thus, individuals are followed for a maximum of four years. We selected 2008 as the baseline year because this corresponded to the marked rise in housing arrears and the onset of the economic recession, and tracked individuals through to 2010. The overall individual non-response rate varies across countries, ranging from around 10% in Cyprus to over 40%in Denmark and Norway.^21^ The framework of the EU-SILC requires countries to use representative probabilistic samples. This is achieved in all of the countries included in our analysis with the exception of Spain, which uses substitution methods to replace non-respondents.^22^ Representativeness was assessed using data from population registers and censuses.^21^ For comparability we constrained the sample to persons aged 16 and over, who reported both being employed and not being in housing arrears in the baseline 2008 survey, and who were present in all three of the surveys. By limiting the sample in this way the analysis was better able to investigate the effects of moving into arrears and/or unemployment on health. We also excluded respondents who lived in homes that were supplied rent free. This resulted in a sample size of 136 371 person/years (45 457 individuals), clustered within 28 416 households in 27 countries. Country sample sizes varied between 294 person/years for Bulgaria to over 14 000 for France and the Netherlands. All countries were retained in order to maximize the country level sample size, included countries are given in Table 1.

**Table 1:** Descriptive statistics for outcome and predictor variables in retained sample

		2008	2009	2010
Health		3.12 (SD 0.75)	3.09 (SD 0.76)	3.07 (SD 0.76)
Housing arrears	No arrears	45 457 (100%)	39 654 (96.3%)	39 991 (96.0%)
	Arrears	0	1508 (3.66%)	1672 (4.01%)
Gender	Male	24 111 (53.0%)	24 111 (53.0%)	24 111 (53.0%)
	Female	21 346 (47.0%)	21 346 (47.0%)	21 346 (47.0%)
Age		41.4 (S.D. 10.1)	42.4 (S.D. 11.0)	43.4 (S.D. 11.0)
Marital status	Married	28 613 (63.0%)	29 067 (64.1%)	29 463 (64.9%)
	Never married	12 607 (27.8%)	11 993 (26.4%)	11 504 (25,4%)
	Separated/divorced	3594 (7.92%)	3706 (8.17%)	3756 (8.28%)
	Widowed	579 (1.28%)	607 (1.34%)	654 (1.44%)
Education level	Primary only	2411 (5.37%)	2359 (5.24%)	2337 (5.19%)
	Secondary only	26 007 (57.9%)	25 872 (57.5%)	25 840 (57.36%)
	Post-secondary	16 472 (36.7%)	16 761 (37.3%)	16 874 (37.5%)
Tenure	Owner–occupier	34 226 (75.3%)	34 864 (76.8%)	35 326 (77.8%)
	Private rent	7967 (17.5%)	7534 (16.6%)	7282 (16.0%)
	Reduced rent	3256 (7.16%)	3021 (6.65%)	2794 (6.15%)
Disposable income (€1000)		43.7 (S.D. 34.8)	44.5 (S.D. 36.0)	44.5 (S.D. 33.1)
Chronic illness at baseline	No	29 658 (80.3%)	–	–
	Yes	7293 (19.7%)	–	–
Limiting illness at baseline	No	32 393 (87.7%)	–	–
	Yes	3760 (10.2%)	–	–
	Yes, strongly limiting	781 (2.1%)	–	–
Economic activity	Employed	45 457 (100%)	41 637 (92.0%)	40 528 (89.6%)
	Unemployed	0	1530 (3.38%)	1812 (4.01%)
	Retired	0	750 (1.66%)	1343 (2.97%)
	Other inactive	0	1312 (2.90%)	1549 (3.42%)

Sample countries: Austria, Belgium, Bulgaria, Cyprus, Czech Republic, Denmark, Estonia, Greece, Spain, Finland, France, Hungary, Iceland, Italy, Lithuania, Luxemburg, Latvia, Malta, The Netherlands, Norway, Poland, Portugal, Romania, Sweden, Slovenia, Slovakia and the UK.

Housing arrears and economic activity other than employed are zero in 2008 due to sample constraints.

Housing arrears were measured using the following question: ‘In the last 12 months, has the household been in arrears, i.e. has been unable to pay on time due to financial difficulties for: (i) rent (ii) mortgage repayment, for the main dwelling?’

The majority of countries coded responses as ‘yes, once’, ‘yes, twice or more’ and ‘no’ for this question from 2008 onwards, however between five and eight countries (depending on the year) collected only binary yes/no responses for some or all of their sample. Thus to maximize inclusion of countries we collapsed the three-category response into the dichotomous variable.

#### The outcome variable: self-reported health

Self-reported health was measured using the question: ‘How is your health in general?’ We recoded this variable so that higher scores correspond to better health (from 0 very bad to 4 very good). Year averages are shown in Table 1. Although self-reported health is subject to perceptual and cultural biases it is a widely used indicator of individual health and has been shown to correlate strongly with mortality in many countries.^27^^,^^28^ The longitudinal design, which compares individual changes within countries in self-reported health, with country-level clustering/random effects, addresses any potential biases arising from cultural and language differences.

#### Statistical models

We use linear regression, multilevel analysis and longitudinal regression. Linear regression is used initially to give a basic idea of the relationship between housing arrears and health. Further models adjust for the nested structure of the data. Initially we conducted linear regression with country dummy variables, followed by multilevel models with within individual, household and country-level clustering, as follows:

Equation 1:

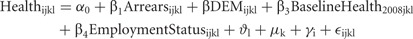

 Here i = year, j = individual, k = household and l = country levels. DEM represents a vector of demographic and other control variables: gender, age, marital status and education level, housing tenure, disposable income and employment status. Baseline health refers to a dummy variable of the presence of chronic illness and health limitations at the baseline year of 2008. γ_i_, μ_k_ and ϑ_l_ are the year, household and country specific random effects while ϵ_ijkl_ is the overall individual error term.

In the final model we further include individual fixed effects in a longitudinal linear regression.

Continuous variables are grand mean centred. All regression models are weighted with standard errors clustered at the country level, with listwise deletion. Multilevel analysis is unweighted as the EU-SILC data do not report conditional weights. Coefficients are standardized.^23^ The models were performed using Stata 13 xtmixed and xtreg functions. Descriptive statistics are given Table 1.

## Results

### The impact of housing arrears on health

The mean self-reported health score over the period of study was 3.09, with a standard deviation of 0.75. In the sample, 2458 individuals (6.15%) transitioned from no housing debt at baseline to experiencing housing arrears between 2008 and 2010.

Table 2 presents the results of seven multivariate models of the impact of transitioning into housing arrears on health. In the first unadjusted model (Model 1), we observed that transitioning into housing arrears was associated with a statistically significant reduction in self-reported health of −0.14 U (95% CI −0.10 to −0.19). This association was consistent, albeit slightly attenuated, after controlling for a range of demographic factors (−0.11, 95% CI −0.06 to −0.16, Model 2). With the addition of variables relating to chronic and limiting illness at the baseline year the coefficient for housing arrears reduces only slightly (−0.09, 95% CI −0.06 to −0.12, Model 3); another slight reduction occurred with the introduction of economic status (−0.07, 95% CI −0.04 to −0.10, Model 4).

**Table 2:** Estimated impact of transitioning into housing arrears on self-reported health, 2008–10, baseline sample of individuals not in arrears and employed, standardized coefficients

Covariate	Model 1	Model 2	Model 3	Model 4	Model 5	Model 6	Model 7
Housing arrears	−0.14^***^	−0.11^***^	−0.09^***^	−0.07^***^	−0.08^***^	−0.05^***^	−0.03^**^
	(0.04)	(0.03)	(0.02)	(0.02)	(0.01)	(0.01)	(0.01)
Age	–	−0.35^***^	−0.38^***^	−0.41^***^	−0.34^***^	−0.36^***^	−0.19
		(0.04)	(0.03)	(0.04)	(0.03)	(0.03)	(0.39)
Age^2^	–	0.11*	0.21^***^	0.24^***^	0.16^***^	0.17^***^	−0.24
		(0.05)	(0.05)	(0.06)	(0.03)	(0.03)	(0.30)
Female	–	−0.04^***^	−0.03^***^	−0.03^***^	−0.03^***^	−0.03^***^	–
		(0.01)	(0.01)	(0.01)	(0.01)	(0.00)	
Marital status							
Never married	–	−0.01	−0.00	−0.01	−0.01	−0.00	−0.02
		(0.01)	(0.01)	(0.01)	(0.01)	(0.00)	(0.01)
Separated or divorced	–	0.00	0.02*	0.02*	0.01	−0.01	−0.04*
		(0.01)	(0.01)	(0.01)	(0.01)	(0.01)	(0.02)
Widowed	–	−0.11^**^	−0.06^***^	−0.06^***^	−0.05^***^	−0.05^***^	−0.01
		(0.03)	(0.01)	(0.01)	(0.01)	(0.01)	(0.06)
Married	–	Ref.	Ref.	Ref.	Ref.	Ref.	Ref.
Education level							
Post-secondary	–	0.23^***^	0.19^***^	0.19^***^	0.14^***^	0.14^***^	0.04
		(0.04)	(0.04)	(0.04)	(0.01)	(0.01)	(0.06)
Secondary	–	0.12^**^	0.10*	0.10*	0.05^***^	0.07^***^	0.02
		(0.04)	(0.04)	(0.04)	(0.01)	(0.01)	(0.06)
Primary	–	Ref.	Ref.	Ref.	Ref.	Ref.	Ref.
Tenure							
Private rent	–	−0.05*	−0.05*	−0.04*	−0.05^***^	−0.04^***^	−0.02*
		(0.02)	(0.02)	(0.02)	(0.01)	(0.00)	(0.01)
Reduced rent	–	−0.06^**^	−0.05^**^	−0.05^**^	−0.05^***^	−0.04^***^	−0.01
		(0.02)	(0.02)	(0.02)	(0.01)	(0.01)	(0.01)
Owner occupier	–	Ref.	Ref.	Ref.	Ref.	Ref.	Ref.
Disposable income (1000s)	–	0.08^***^	0.07^**^	0.07^**^	0.05^***^	0.04^***^	−0.001
		(0.02)	(0.02)	(0.02)	(0.01)	(0.00)	(0.002)
Chronic Illness at baseline year (yes)	–	–	−0.28^***^	−0.28^***^	−0.29^***^	−0.27^***^	–
			(0.02)	(0.02)	(0.02)	(0.01)	
Limiting illness at baseline year							
Yes, strongly limiting	–	–	−0.51^***^	−0.50^***^	−0.50^***^	−0.53^***^	–
			(0.03)	(0.01)	(0.02)	(0.01)	
Yes	–	–	−0.26^***^	−0.26^***^	−0.26^***^	−0.26^***^	–
			(0.01)	(0.01)	(0.01)	(0.01)	
No	–	–	Ref.	Ref.	Ref.	Ref.	Ref.
Economic activity							
Lost job	–	–	–	−0.09^***^	−0.08^***^	−0.06^***^	−0.02^**^
				(0.01)	(0.01)	(0.01)	(0.01)
Retired	–	–	–	−0.03	−0.02	−0.03^**^	0.02
				(0.03)	(0.03)	(0.01)	(0.02)
Other inactive	–	–	–	−0.10^**^	−0.12^***^	−0.08^***^	−0.02
				(0.03)	(0.02)	(0.01)	(0.03)
Employed	–	–	–	Ref.	Ref.	Ref.	Ref.
Variance parameters							
Country (SD)	–	–	–	–	–	0.04	–
						(0.01)	
Household (SD)	–	–	–	–	–	0.09	–
						(0.00)	
Individual (SD)	–	–	–	–	–	0.08	–
						(0.00)	
Residual (SD)	–	–	–	–	–	0.24	–
						(0.00)	
*R*^2^	0.002	0.09	0.23	0.23	0.26	0.23	0.06
Country level random effects	No	No	No	No	Dummy variables	Yes	No
Household random effects	No	No	No	No	No	Yes	No
Individual random effects	No	No	No	No	No	Yes	No
Individual fixed effects	No	No	No	No	No	No	Yes
Number of individual-years	n/a	97 811	97 145	96 864	96 864	101 155	99 916

*Notes:* Data from EU-SILC 2010 longitudinal dataset. Robust standard errors clustered at varying levels with each model. Age squared divided by 100 to facilitate interpretation. Coefficients standardized following Gelman (2008) who advocates dividing by two standard deviations, variance parameters for multilevel model taken from unstandardized outcome model.

*P < 0.05; ^**^P < 0.01; ^***^P < 0.001.

Consistent with previous research, we found that unemployment was associated with an independent deterioration in health (−0.09, 95% CI −0.07 to −0.11, Model 4), similar in magnitude to arrears, evidence for cumulative disadvantage.^24^^,^^25^ We also observed patterns in our models that have been reported elsewhere: women are more likely to report worse self-reported health (although this is in part due to differential survival^26^), and age is associated with lower self-reported health. Those who are married report better health than those who have been widowed, but do not differ significantly from those who were never married. Higher levels of education were associated with higher levels of health, as was increased disposable income. In terms of housing status, renters report poorer health than owner occupiers, irrespective whether at renting at market (−0.04, 95% CI −0.01 to −0.08) or reduced rate (−0.05, 95% CI −0.02 to −0.09, Model 4).

We next included country dummies (Model 5), which account for potential cultural differences.^27^^,^^28^ The coefficient for housing arrears increased slightly (−0.08, 95% CI −0.06 to −0.09). Then, to account for changes within individuals over time, we conducted a multilevel analysis including household and individual as well as country random effects (Model 6). The coefficient for housing arrears is again slightly attenuated (−0.05, 95% CI −0.03 to −0.06), but remains statistically significant (*P *< 0.001). Finally, in the most conservative model (Model 7) we re-estimated the model but included individual fixed effects, utilizing only variation within-individuals over time, finding consistent results for the impact of housing arrears (−0.03, 95% CI: −0.01 to −0.04).

### The modifying role of housing tenure

**Figure 1:**
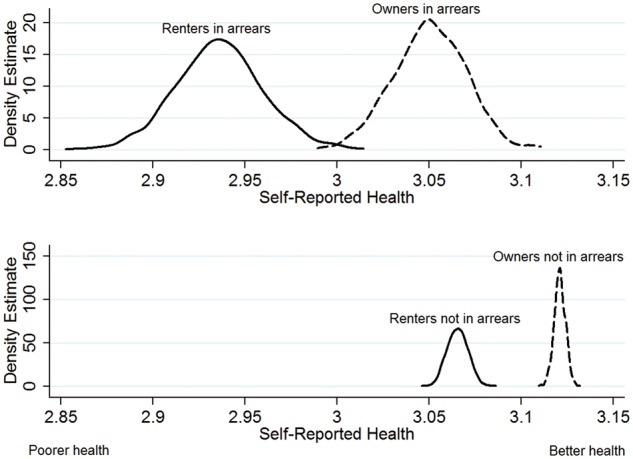
Interaction of housing tenure with housing arrears. Notes*:* Estimates based on Stata Clarify Monte Carlo 1000 simulations, displayed as kernel density plots. Unweighted regression model with country dummies and standard errors clustered at the individual level. Clarify does not currently support xt functions or weighting

In the final step, we tested whether persons living in rented accommodation would be more adversely affected by falling into housing arrears than owners. We added an interaction between housing tenure and housing arrears to a country fixed effects model based on Model 5 with market-rate and reduced rent grouped together (both variables are time-varying). We found that persons experiencing housing arrears while living in rented accommodation had worse deterioration in self-reported health than owners. Using the CLARIFY module in Stata, performing Monte Carlo simulations, we plotted the interaction term, showing a mean 0.11 unit additional drop in health for persons living in rented housing compared with owner-occupiers (figure 1). The cumulative disadvantage experienced by those in rented accommodation experiencing arrears vs. owner-occupiers who were not in arrears was substantial (mean self-reported health 2.94 vs. 3.12)

We also briefly investigate the effects of housing arrears and tenure on health according to income. We compared the impact of arrears on people according to disposable income quartiles (Table 3) finding that housing arrears had a statistically significant impact on health for all but the very richest (across all tenures), who likely have wealth and income to rely on, and the very poorest owners.

**Table 3:** Estimated impact of transitioning into housing arrears according to household income

	Below 25th percentile	25–50th percentile	50–75th percentile	Above 75th percentile
Housing arrears (all)	−0.05***	−0.05**	−0.08***	0.00
	0.01	0.02	0.02	0.02
Housing arrears (owners)	−0.03	−0.04*	−0.05*	0.02
	0.02	0.02	0.02	0.02
Housing arrears (renters)	−0.06**	−0.07*	−0.14***	−0.06
	0.02	0.03	0.04	0.05

*Note*: Based on Model 6, including all control variables. Renters include both private and reduced rent tenants.

#### Robustness tests

We conducted further tests of our model sample and specification. First we included a measure of the ‘financial burden of total housing cost’, finding no change in the coefficient for housing arrears, although self-reported financial burdens were associated with poorer self-reported health (Web Appendix). Similarly we checked the robustness of our housing arrears measure by running models where only those who reported multiple housing arrears (i.e. those who responded ‘yes, twice or more’ to the housing arrears question) were coded as in arrears; this reduced the sample size due to the limited number of countries using the question in this form. The results of the model were broadly unchanged using this method, with the alternative coding of the housing arrears variable also statistically significant. As a final specificity test, we constructed an alternative baseline sample using those who were in housing arrears at baseline and moved out to see whether health status improved to further confirm that the state of housing arrears is responsible for the decline in health. We find finding a similar association as when persons transitioned into housing arrears.

## Discussion

Our analysis yields three major findings. First, we demonstrate that falling into housing arrears is associated with a statistically significant deterioration in self-reported health, even after correcting for baseline health, potential socio-demographic confounders and other economic difficulties. Second, these associations were in addition and similar in magnitude to those linked to unemployment, which provides evidence of an accumulation of health disadvantage.^29^ This finding shows that the health effects of housing arrears should not be dismissed as the side effect of other financial difficulty. Third, the impact was the greatest in persons who were living in rented accommodation. In sum, these findings demonstrate the importance of financial aspects of housing to health and the likely considerable impact of the recent housing crisis on the health of people living in Europe.

Our study has several important limitations. First, it does not seek to study comprehensively the multifactorial ways that housing can affect health. Thus we do not consider the quality of housing in our models, instead focusing on the effects of arrears. A second limitation is that our study cannot fully ascertain the pathways that lead to arrears. Given the complexity of this phenomenon, this may be best addressed by qualitative research. One example of this approach, albeit with those who have become homeless rather than falling into arrears, was conducted in the Netherlands.^30^ This found that, among 120 recently homeless adults, of whom almost 9 in 10 were male and half were Dutch, the commonest pathways were evictions, relationship problems, and prisons, but there were significant differences in the demographic characteristics of those following the different pathways. Another study from the UK found that homelessness was often the final result of a complex series of experiences, some with roots in childhood.^31^ Clearly, not all those in arrears will lose their homes so the findings cannot be applied directly to the issue of housing arrears but the methods used would be applicable.

A third limitation of this study is that the measure of housing arrears used is unable to capture the severity or length of arrears, nor the full consequences of arrears for the respondent. It was not possible in this data (without a considerable detrimental impact on sample size) to investigate whether respondents were in arrears by single or multiple payments, or whether their status resulted in repossession or eviction, and indeed panel data such as EU-SILC are likely to underestimate the adverse impact of arrears on health as those who become homeless will usually be lost to follow up. Some, but not all studies of those who have lost their homes in various European countries show very high levels of mental illness,^32^ although it is difficult to assess the direction of causality and a recent systematic review cautioned against the assumption that mental illness precipitates homelessness.^33^ Our study, while only addressing the first stages of a possible pathway to homelessness, provides support for the view that the converse association applies to at least some extent.

This article does, nonetheless, contribute towards understanding the impact of housing arrears on health, demonstrating not only its significant negative impact but also the relevance of housing tenure. Those who live in rented homes experience a greater worsening of health relative to those living in owner occupied housing when facing housing arrears. This negative effect on health is comparable to that of becoming unemployed which is significant given the attention paid to unemployment on health, and given that housing arrears does not itself necessarily capture the worst aspects of housing insecurity: eviction or foreclosure. It is likely therefore that the spectrum of housing problems may have even greater impact on health than unemployment.

The finding of a significant health impact of housing arrears is especially important to inform contemporary debates in many European nations in the context of recession and austerity. We estimate an additional 3.5 million persons across Europe experiencing housing arrears in 2010 relative to 2008. This makes arrears a significant feature of the European financial crisis, and the element that has the greatest and most immediate impact on people’s security. The threat arising from housing insecurity and loss is often likely to be greater than that from employment insecurity and loss.

Although our data cover only until 2010, the situation in some countries may now be worse. Recent policy changes in the UK, notably the housing benefit changes associated with the 2012 Welfare Reform Act (often referred to as the ‘Bedroom Tax’), have been associated with considerable increases in housing arrears and homelessness.^34–37^ 29% of Housing Associations reported increases in housing arrears which they attributed to the ‘Bedroom Tax’.^38^ Consequently, the current British government’s welfare policies, including its benefit cap, are likely be deleterious to health, particularly among those living in expensive areas such as London.^39^ In Spain too, support for publicly supported housing (Vivienda de Protección Oficial) has been significantly scaled back in recent years.

Our findings support recent calls by the Joseph Rowntree Foundation in the UK to find alternative means to alleviate pressure on health services at a time when they are under considerable strain.^40^ Reducing the number of people falling into housing arrears may help to reduce the strain on health services, for example. However, these findings have much wider relevance and should stimulate a renewed attention to the issue of housing by public health authorities in all countries.

Key Points

- Transitioning into housing arrears is associated with deterioration in self-reported health, even after correcting for baseline health, potential socio-demographic confounders and other economic difficulties.
- The impact of housing arrears on health is similar in magnitude to unemployment suggesting that housing difficulties should be receive equivalent attention of job loss from health policy researchers and practitioners.
- The impact is greatest for those renting.
